# Comments on “ChatGPT’s role in alleviating anxiety in total knee arthroplasty consent process: a randomized controlled trial pilot study”

**DOI:** 10.1097/JS9.0000000000002390

**Published:** 2025-04-18

**Authors:** Xu Cai, Bolun Wang, Lin Cheng, Qifeng Ou

**Affiliations:** a Emergency Medical Center, The First Hospital of Hunan University of Chinese Medicine, Changsha, Hunan Province, China; b College of Physical Education, Woosuk University, Jeollabuk-do, Korea; c Regenerative Medicine Institute (REMEDI) at CÚRAM SFI Research Centre for Medical Devices, School of Medicine, College of Medicine, Nursing and Health Sciences, University of Galway, Galway City, Galway Country, Ireland

*To the Editor*,

We read with great interest the article by Wenyi *et al*^[^1^]^, in which a randomized controlled trial pilot study was conducted to validate the positive role of ChatGPT in alleviating anxiety during the informed consent process for total knee arthroplasty (TKA). We would like to offer some comments.

First, the experience level of the surgeons involved in patient consultations is a critical factor. In the study, ChatGPT was queried by the surgeon, and its responses were evaluated before being disclosed to the patients. In this context, a surgeon’s level of experience may influence how they interpret ChatGPT’s responses. Were they residents, fellows, or experienced attending physicians? Given this approach, the randomization and control of selection bias should focus on the surgeon rather than the patients – specifically, on the surgeon’s ability assess ChatGPT-generated responses.

Additionally, it would have been valuable to include a control group in which surgeons were allowed to use Google (or similar search engine) for comparison. Google’s search algorithm personalizes results based on an individual’s search history, potentially providing more tailored and contextually relevant information. This contrasts with ChatGPT, which generates standardized responses independent of user-specific profiles^[^2^]^.

Another consideration is whether patients in the intervention group were allowed to use ChatGPT independently outside the consultation. Patients and their families in this group, having witnessed the use of ChatGPT during the consultation, were more likely to recognize its potential in addressing TKA-related questions. This awareness could have led them to independently seek AI-generated information, thereby introducing bias in the perceived efficacy of the surgeon’s use of ChatGPT in alleviating patient anxiety.

Also, regarding frequently asked questions – such as the incidence of postoperative infection – the reported rates may vary between surgical teams and depend on the patient’s health status, particularly factors such as diabetes, obesity, and under immunosuppressive therapy. Usually, an experienced surgeon can provide personalized responses based on the patient’s overall condition and their own clinical experience with similar cases. At present, AI models may lack the ability to generate such tailored responses until sufficiently trained prognostic models are developed.

Last, using an AI model during consultations may introduce distrust between patients and doctors, as such behavior could give the impression that the doctor is overly reliant on AI, appearing less professional and less experienced. Caution should be taken when using AI models during the consultation process for informed consent.

## Data Availability

Data sharing is not applicable to this article.
